# State-of-the-Art Wearable Sensors and Possibilities for Radar in Fall Prevention

**DOI:** 10.3390/s21206836

**Published:** 2021-10-14

**Authors:** José Gabriel Argañarás, Yan Tat Wong, Rezaul Begg, Nemai Chandra Karmakar

**Affiliations:** 1 Electric and Computer Systems Engineering Department, Monash University, Clayton, VIC 3800, Australia; yan.wong@monash.edu (Y.T.W.); nemai.karmarkar@monash.edu (N.C.K.); 2 Physiology Department, Monash University, Clayton, VIC 3168, Australia; 3 Institute for Health & Sport, Victoria University, Melbourne, VIC 3032, Australia; rezaul.begg@vu.edu.au

**Keywords:** wearables, radar, wireless, smart shoes, sensor network, gait analysis, fall prevention

## Abstract

Radar technology is constantly evolving, and new applications are arising, particularly for the millimeter wave bands. A novel application for radar is gait monitoring for fall prevention, which may play a key role in maintaining the quality of life of people as they age. Alarming statistics indicate that one in three adults aged 65 years or older will experience a fall every year. A review of the sensors used for gait analysis and their applications to technology-based fall prevention interventions was conducted, focusing on wearable devices and radar technology. Knowledge gaps were identified, such as wearable radar development, application specific signal processing and the use of machine learning algorithms for classification and risk assessment. Fall prevention through gait monitoring in the natural environment presents significant opportunities for further research. Wearable radar could be useful for measuring gait parameters and performing fall risk-assessment using statistical methods, and could also be used to monitor obstacles in real-time.

## 1. Introduction

The concept of successful ageing has been gaining increasing attention in recent years, and is key to achieving long-lasting quality of life. This concept can be understood as a combination of avoiding disease and disability, maintaining high cognitive and physical function and engagement with life [1,2]. However, one in three adults aged 65 or more will experience a fall every year [3,4]. This represents a major threat to successful ageing and independent living due to acute or chronic pain, fear of falling and serious injuries [5].

According to the World Health Organization, falls are the second largest cause of unintentional injury resulting in death [6]. Each year, more than 600,000 people worldwide die of fall-related injuries [6]. Associated costs are also a significant issue and, as average age is rising [7], the cost of falls is projected to increase to US $240 billion by 2040 [3].

Wearable devices have been revolutionizing the biomedical field in the last three decades and have led to concurrent advances in gait analysis [8,9,10]. Inertia measurement units (accelerometers, gyroscopes and magnetometers), ultrasonic sensors and force and pressure sensors are among the most widely used and thoroughly researched biomedical devices. The use of radar sensors as wearables, however, remains largely uncharted, though references to their potential have been emerging in the literature in the past five years.

Fall detection has been thoroughly researched in the past decades and focuses on detecting a fall that has already occurred, enabling first responders to respond quickly, and improving the chances of patient recovery. On the other hand, fall prevention aims to avert the fall from occurring in the first place, and has received less attention in the literature. This paper will focus primarily on reviewing gait monitoring and fall prevention, and the sensors that can be used for such an end.

Fall prevention research focuses on identifying and controlling risk factors for falls [11,12]. These risk factors can be broadly grouped into extrinsic and intrinsic factors, and both groups feature both controllable and uncontrollable factors [13]. Extrinsic factors are related to the individual’s surroundings, and some of these are controllable, for example footwear and features of the home-environment, including secure floor coverings and non-slip surfaces. Other factors are, however, uncontrollable, such as features of the outdoor environment, including uneven footpaths, obstacles, surface–height negotiation and slippery surfaces [14]. Intrinsic factors include age, gender, medical conditions and falls history. Controllable intrinsic factors can be medications, visual abilities, cardiovascular status, gait and balance.

Analysis of risk factors identifies gait and balance as key opportunities for fall prevention [15]. While external and uncontrollable risk factors are beyond the scope of patients and clinicians to exhaustively anticipate and manage, intrinsic and controllable factors represent a strategic target for interventions that can optimize patient awareness and avoidance of risks. Significant progress has been made in controlling some of the intrinsic risk factors mentioned above, with physicians routinely addressing medication [16], visual ability [17], exercise and muscular and cardiovascular status [18]. However, gait and balance have traditionally been considerably more difficult to monitor and manage outside laboratory settings [19,20]; thus, there is a significant gap in fall prevention that can be addressed using interventions enabled by advances in wearable sensor technologies [21]. Approximately 30% of falls occur during locomotion [22], and a survey study found that 53% of fall patients reported the cause of their fall to be tripping [23]; therefore, targeting the gait pattern of patients has the potential to address a large percentage of fall-related incidents and will have a significant impact on reducing falls [24].

This paper is structured as follows: firstly, gait analysis and fall prevention are introduced. Then, state-of-the art wearable devices are presented, continued by a specific review of shoe-mounted wearable devices. Later, the use of radar in gait analysis and falls is described, starting with the classical approach of non-wearable radar as a replacement of motion capture systems and moving on to the more novel wearable radar, including their limitations and challenges, identifying a gap in the research. Next, a proof of concept of wearable radar for obstacle detection is presented, which could be used to achieve fall prevention to fulfill this gap. Finally, emerging applications, conclusions and recommendations for future work are outlined.

## 2. Gait Analysis and Fall Prevention

Gait analysis is the systematic study of human movement during locomotion [25]. For more than a century, it focused on studying the gait cycle and its parameters. The gait cycle is defined as the interval on which one limb goes from a first heel contact to the next heel contact [26]. This cycle can be divided in two major stages: stance and swing. Stance is the period in which the limb is in contact with the ground, and it takes ~60% of the gait cycle time. Swing is the phase where the limb is moving without having contact with the walking surface, and it takes ~40% of the time. The most common gait cycle parameters that were traditionally considered include spatial and temporal parameters such as stance/swing times and step/stride lengths, toe-angle and swing foot trajectory parameters such as minimum foot clearance [26,27].

More recently, gait analysis has been extended to include a broader classification of locomotor and postural activities, e.g., walking, running and sitting [28,29,30]. Systems for gait analysis can be divided into three major groups: Non-Wearable Systems or context-aware systems (NWS), Wearable Systems (WS) and Combined Systems or fusion systems (CS) [11]. These three categories can be sub-divided by the methods used to obtain the data, e.g., 3D motion capture, digital video recording, accelerometers and radar.

**Figure 1:**
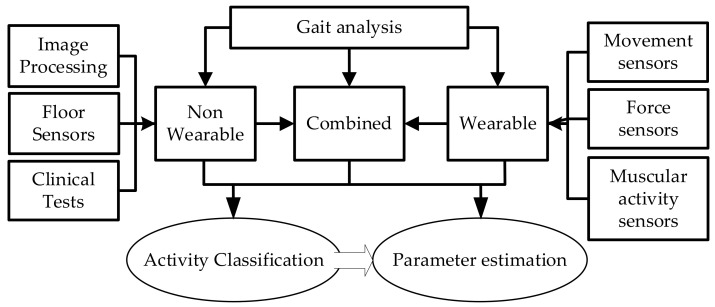
Classification of gait analysis systems. Gait analysis can be achieved using non-wearable and/or wearable systems. Both systems can achieve similar types of results but with significant differences in price, usability, resolution and accuracy.

**Figure 2:**
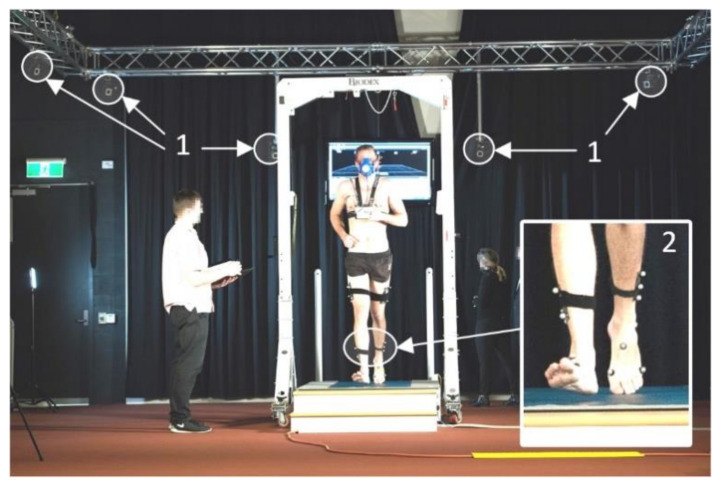
Gold-standard gait lab setup. The participant is walking on a force plate integrated into an AMTI treadmill. Vicon motion capture cameras fixed on a truss (1, arrows) track the location of reflective markers (2) mounted on participant’s foot and shank. This complex system can calculate body segments’ movements with sub-millimeter accuracy and synthesize force vector through the recorded triaxial ground contact forces. Photo courtesy of Victoria University Gait Laboratory.

There are three main categories of non-wearable systems, which are based on Image Processing, Floor Sensors [31] or quantitative clinical testing (Figure 1). These systems are laboratory-based and are currently the gold standards for gait analysis. Motion Capture Systems (MCSs) use an array of high-speed cameras to determine the position of reflective markers attached to the subject’s body through image processing (Figure 2).

Later, by tracking the position of these markers, it is possible to obtain gait parameters with submillimeter accuracy [32]. MCSs are often combined with force platforms to obtain the ground reaction forces in three axes [33], allowing for a more complete picture of the subject’s gait characteristics. Clinical testing methods combine observational gait analysis with the measurement of a variable such as walking speed using a stopwatch. The main advantages of these methods are that they are very low-cost and can be performed in hospital rooms and caregiving facilities without specialized equipment. Some examples include the Timed Up and Go test [34] and the Four Square test [35]. Wearable sensors, which were first introduced in 1989 by Péruchon et al. [36], will be discussed in detail in Section 3.

Fall prevention has become one of the most important applications of gait analysis due to the high frequency and cost of falls, particularly among the elderly [37]. Stiffness, lack of coordination, impaired reflexes, reduced muscle strength and tone, shorter step length and height and reduced ability to take corrective action after an unforeseen trip or slip are among the causes of gait-related falls [4,14,37]. To address these causes, studies have developed methods to quantify and classify an individual’s risk of falling. For example, Begg et al. [22] focused on minimum foot-ground clearance during the swing phase to predict the risk of falling. Di et al. [38] developed a motorized robot cane to provide guidance and obstacle avoidance using a laser range finder, and fall detection and prevention by means of an algorithm that combines the estimation of the user’s center of gravity with the position of the cane. Majumder et al. [39] predicted falls using smartphone accelerometers and smart shoe insole pressure sensors. The combination of data from these two sensors triggered an alarm when gait abnormalities were detected. Several studies have investigated the subjects’ functional ability and behavior with different technologies, such as RFID and nonlinear classification, to prevent falls in constrained environments such as acute care facilities [40,41,42].

Ongoing challenges in fall prevention research include the large number of uncontrollable risk factors, such as slippery or unstable surfaces, neurological function, chronic medical problems and previous fall history [13,14], achieving user acceptance for preventive devices [43] and, in some cases, the lack of reliable sensors and methods [44]. With further work to address these challenges, wearable sensor devices offer significant opportunities for gait analysis in fall prevention by providing quantitative data for reliable patient monitoring and the accurate prediction of falls.

It is clear that the role of gait analysis in fall prevention is appreciable [45], and has gained importance through the application of wearable sensors in real-world environments such as homes, offices, sports fields and public spaces, and this will be the subject of the next section.

## 3. Wearable Devices

**Figure 3:**
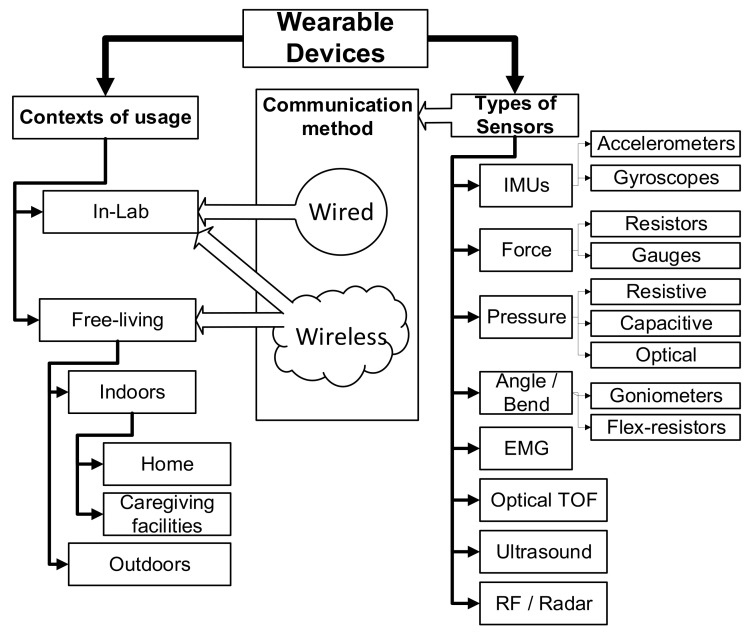
Classification of Wearable Devices. This classification shows how the different types of sensors can be used in different contexts depending on the communication method. TOF stands for time-of-flight, EMG for electromyography and IMUs for inertia measurement units.

Wearable electronic devices for gait analysis have been studied extensively over the past three decades [19,31,44,46,47] and a broad overview is provided in order to contextualize the discussion. Wearables can be used in two different contexts, in-lab and free-living (see Figure 3). In the laboratory, wearables are used when performing predetermined tasks, such as walking on a treadmill, often combined with non-wearable methods such as motion capture, force plates and video recording. The second and most interesting use of wearables is in uncontrolled free-living gait analysis [21]. Although such analysis can provide information that would not be possible in a laboratory, the continuous data provided by sensors necessitates differentiation of activities in order to be able to extract and interpret gait parameters [44]. Extensive work has addressed this issue. For a detailed list of methods see Table 1 of [44].

**Figure 4:**
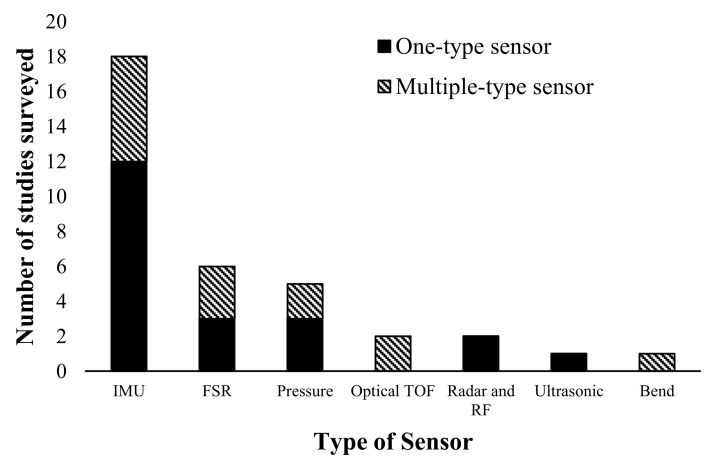
Popularity of types of sensors used in shoes in the literature reviewed. Solid bars show the number of times that the sensor type was used on its own. Diagonal-pattern bars show the number of times that the sensor type was used in combination with other types of sensors. Inertial measurement units (IMUs) are the most popular sensor and have been extensively explored due to their miniature size and negligible cost. Displacement and velocities can be calculated by integrative methods, although the results have limited accuracy and suffer from drift.

Numerous studies have focused on sensors to characterize foot motion for the determination of gait parameters (summarized in Figure 4). Although each sensor type has its strength, significant weaknesses affect each sensor’s utility (Table 1). Among these studies, several have utilized data from shoes to predict the risk of falling and prevent falls by providing feedback alerts to the user and notifying caregivers or clinicians. These studies have explored a range of sensor types, the most common of which are outlined below.

Inertia Measurement Units (IMUs) are the most popular sensor, given that they are easy to work with and inexpensive. They are comprised of accelerometers and gyroscopes packaged into small chips (3 mm × 3 mm × 1 mm), making them an excellent device for embedding into shoes. Three-axis accelerometry and gyroscopic data can be obtained to estimate spatiotemporal parameters, and can be further processed using integrational methods to estimate specific biomechanical variables such as foot clearance, although these methods have limited accuracy. Inertia measurement units are often combined with other sensors to improve accuracy or estimate parameters that cannot be obtained from accelerometry. In the fall prevention field, IMUs have been used to predict abnormal gait patterns by using different classification techniques such as K-nearest neighbor and support vector machines. A further drawback of IMUs is drift, which can present a major problem when extracting data over long periods of time.

The second most common sensor type comprises Force Sensitive Resistors (FSRs), which are used to measure ground reaction forces by installing them inside or below the soles of footwear. FSRs are inexpensive and provide good estimates of temporal parameters such as step time, stride time and stance time [8,9,48,63,64,65,66,67]. Pressure sensors are often used in place of FSRs for similar purposes and they also provide heatmaps of the user’s footprint. Studies have investigated using pressure sensor data to estimate spatiotemporal gait parameters, study foot problems such as diabetic foot and deformity and to estimate fall risk [39,51,56,59,68,69,70].

Minimum foot clearance is a gait parameter of particular interest in fall prevention because of its correlation with tripping risk [22,75]. Various sensors have been explored to measure the parameter directly, namely ultrasonic, Optical Time-Of-Flight (OTOF) and radar. Ultrasonic sensors act as sonars and they can be used for estimating the distance between the floor and the subject’s foot [49,50]. They have good accuracy and are inexpensive but are oversized for the application (40 mm × 20 mm × 20 mm) and must be externally mounted on the shoe. Another disadvantage of ultrasonic sensors is that they usually consume more DC power than RF sensors, since they have to physically push–pull, or vibrate a structure to generate the ultrasonic waves [76]. OTOF sensors [61,62] are accurate, lightweight and very small (5 mm × 3 mm × 2 mm), making them highly suitable for embedding in shoes. They operate well under most conditions, including black surfaces, which cause only a loss of precision due to their low reflectivity of light for this sensor type.

Radar sensors have also been explored [71,72,73] and provide a good method for measuring foot clearance. These sensors have the additional capability of scanning the environment, which will allow significant advances in fall prevention by enabling obstacle detection. Millimeter-wave technology has evolved to off-the-shelf full system-on-chip (SoC) radars, which have the potential to enable the development of a new generation of environment scanning devices for fall prevention. In this regard, a system such as smart shoes [70] would be instrumental in facilitating gait monitoring for fall prevention by providing data on parameters such as foot–ground clearance [45], stride time, gait speed and left–right symmetry [77]. The patient can then be provided with crucial information about their gait, and this could lead to intervention by caregivers or clinicians as necessary [78]. Wearable radar can be a significant addition to the collection of wearable sensors commonly used in monitoring and recording devices in this field, adding the capability of detecting obstacles in the environment. Despite the great potential advantages that this technology can bring, there is still a gap in research and development to achieve the needed technology readiness.

## 4. Radar in Gait Analysis and Fall Prevention

The term RADAR was coined in the early 1940s as an acronym for RAdio Detection And Ranging. Radar detects objects by emitting radiofrequency waves and analyzing the reflected signals. It can also determine the position and velocity of objects depending on the type of signal emitted, e.g., pulsed or continuous-wave, and on the processing applied to the return, e.g., moving-target indicator, constant false-alarm rate and/or Doppler. Applications of radar are numerous, including air-traffic control, surveillance, weapons fire control, weather prediction and vehicle speed detection. In the last decade, gait monitoring has emerged as a new application of radar, because it presents an affordable alternative to motion capture systems and overcomes privacy issues while providing accurate enough measurements.

This section reviews this work, classifying radars, outlining their benefits and suggesting future gait monitoring possibilities. Table 2 lists the works reviewed in this section and provides a summary of their results. In Section 4.1, we provide a brief review of the classic use of radar as a non-wearable system and as an alternative to cameras or other fixed position sensors. In Section 4.2, we present a novel use of radar as wearable devices, enabled by the miniaturization of mm-wave systems.

### 4.1. Non-Wearable Radar Systems

Many researchers have studied non-wearable radars to provide alternatives to Motion Capture Systems (MCSs), given that they are simpler, less expensive and protect patient privacy by not capturing video [97]. Simplicity comes from being comprised usually of just one device with two or more antennas, whereas motion capture systems require a fixed array of specialized cameras plus considerable processing power in the computer receiving the images from the cameras. In addition, the subject is required to wear reflective markers (passive or active) for motion capture systems (see Figure 2). A further advantage of non-wearable system (NWS) radar over MCSs is price; a complete system-on-chip or system-on-board radar can be purchased for under US $700 [98]. Continuous-Wave (CW) Doppler radar has been used to extract gait parameters using short-time Fourier transforms (STFT) and chirplet transforms, which have been shown to operate well without concerns due to illumination, clothing, occlusion or and weather [79]. Radars can also track objects from long to short range and estimate some gait parameters from 120 m [79].

Some research explored the possibility of automatically detecting falls indoors by analyzing the Doppler signature of the radar return offline and achieved a good classification rate (between 0.91 and 0.97) [81,82]. Commercial applications are offered using radar as the primary fall detection sensor [99]. Other works studied the feasibility of pulse-Doppler radar for estimating gait parameters that could be input into a fall risk assessment and concluded that radar was a viable candidate for the task. The parameters measured included stride variability, although accuracy was low [83], and gait speed [84], which was also identified as needing further work [84,85]. Other works combining radar, wireless communications and data-processing techniques reported up to a 94.3% success rate in detecting fall-like events [87].

Standard CW Doppler radar systems are potentially useful for gait analysis applications but present some problems. The first is that the Doppler return is dependent on the relative direction of motion of the target with respect to the radar’s antenna, and standard CW radar is unable to detect direction of arrival, making the estimation of gait parameters complex [97]. Second, accuracy is low due to factors such as reference stability and environmental clutter. One study found that they could only achieve a maximum accuracy of 80% even when the subject was walking straight towards the radar [88]. This could be overcome by implementing a correction factor calculated as a function of the angle between the trajectory of the walk and the radar’s radial direction, which might be achieved using techniques such as Frequency-Modulated Continuous-Wave (FMCW) [100] and Multiple-Input Multiple-Output (MIMO) [101] to obtain range, elevation and azimuth measurements [98].

Most studies of non-wearable radar with gait analysis/monitoring as the principal aim have been based on Doppler, CW and pulse radar, and most of these focused on fall detection. An extensive review of fall detection can be found in [102]; the present work was designed to review the use of radar for fall prevention. Only one paper where non-wearable radar was advertised as being used directly for fall prevention was found [97]; however, this paper only estimated gait velocity and there was no fall prevention or fall risk assessment algorithms. There have been some reports of using radar for gait parameter estimation such as gait speed and step length, which can be used as indicators of falls risk that can then be used by clinicians to prescribe fall preventative measures in the home such as special footwear, exercising, furniture rearrangement and installing non-slip surfaces, among others. All of these reports emphasized the potential of non-wearable radar for fall prevention through risk assessment, but identified drawbacks, such as being limited to confined spaces (bedrooms, living rooms or hallways in homes or caregiving facilities) and being unable to distinguish specific individuals, which makes it difficult in a multi-occupant home situation [89], without extra sensors such as cameras, to provide identification of patients. A further challenge for radar-based systems is clutter [90,91], given that in a daily living environment many other objects are present. Even though most clutter will provide stationary signal returns and be easily removed by low-pass filtering zero Doppler-speed, moving objects (pets, other people and machinery) will produce competing signal returns that will be difficult to filter out [92]. This is an important area requiring further research.

### 4.2. Wearable Radar Systems

Radar systems can be adapted to a wearable form using System-on-Chip (SoC) devices [98], particularly ones operating in millimeter wave domains due to their small size. A small number of studies have been conducted on wearable radar technology for fall prevention, and their results are promising. They have shown that this technology could be used for many applications, such as calculating foot clearance, either by measuring the return time of flight of the radar signal [71], using DFCW (dual frequency continuous wave) techniques or measuring the distance from shoe-to-shoe to estimate stride length [72]. Radar could also offer new possibilities in fall prevention by enabling the detection of obstacles in the walking path as shown in [73,94,96], and, at the same time, measuring gait parameters to reveal the lower limb control strategies required to safely negotiate obstacles in the everyday environment.

Even though wearable radar and wearable devices in general have advantages over non-wearable systems, user acceptance can be a barrier when the device is to be worn long-term [43]. In the case of wearables for fall prevention, the mode by which information is presented to the user is key to the device’s effectiveness [103]. There is an exciting opportunity in wearable radar for fall prevention to develop embedded antenna systems, smaller form-factor radar systems and information extraction algorithms to inform the user in real time via an engaging user interface.

Most of the studies surveyed presented solutions with large bandwidths (>4 GHz), with range resolutions of 7 to 100 mm. However, several studies presented a single-input single-output approach that is only able to detect one dimension or do Inverse Synthetic Aperture radar (ISAR) through acquiring several frames by moving the target to obtain a 2D image. Others presented a small MIMO approach with only a few Transmit–Receive elements that achieved 2D location of targets. However, most studies did not report their cross-range resolution other than [104], who reported 15 cm in 1 m. Of particular interest is a study that introduced a scalable RF radar frontend in 79 GHz that would enable the development of massive MIMO [105] that is yet to be explored for this application. This SoC requires external antennas and a Local Oscillator (LO) and can be cascaded or stacked to significantly simplify the design of the LO distribution network.

A number of research challenges remain for achieving reliable obstacle detection and measurement of gait parameters. One of these challenges is the detectability of everyday objects that are likely to act as obstacles. The detectability of an object is proportional to its radar cross-section (RCS), which depends on its shape, size and material composition [106]. Conductive objects are the easiest to detect; however, everyday objects are usually made of plastics, wood and other non-conductive materials presenting lower RCSs, making detection more difficult. RCSs also depend on the operating frequencies used: the higher the frequency the more sensitive the radar can be to non-conductive materials, as well as the higher the BW that can be used. Other challenges include achieving good range resolution depending on the radar technique used (i.e., directly proportional to BW in FMCW), and the azimuth and elevation resolutions and accuracies, which depend on the number of elements and shape of the array in MIMO approaches.

## 5. Wearable Radar for Fall Prevention: Proof of Concept

As indicated earlier, tripping over obstacles is a major cause of falls [17,107]; thus, a system that can perform early detection of objects in the predicted walking path could prove highly effective in preventing falls. Advancements in mm-wave technology have allowed the miniaturization of radar systems to enable wearable devices that can be fully contained within a shoe.

To our knowledge, only a few works presented research in this field. Tang et al. focused on detecting a metallic can with a shoe-mounted 24 GHz radar, showing that detection and location in 2D of the can was possible, but did not provide numerical results on its performance [73]. Later on, they expanded on this system, presenting some distance measurements to a target in [94,96].

Shoureshi et al. presented a multi-sensor system that included a FMCW radar on a belt; however, they provide very little information about it, focusing more on showcasing their vibratory feedback system integrated into a vest for visually impaired users [95].

Preliminary laboratory trials were conducted in an anechoic chamber by Argañarás et al. in order to test the feasibility of non-conductive obstacle detection using mm-wave 60 to 64 GHz radar, presenting promising results [108,109].

In this section, we will expand on those findings by detecting non-conductive objects in a real-world setting. This represents significant progress in this field, given that most everyday obstacles are made of non-conductive materials. Three trials were conducted, one mounted on a stand and two mounted on a subject’s leg. The radar was a Texas Instruments IWR6843ISK [110] + MMWAVEICBOOST [111], connected to a laptop computer through USB.

### 5.1. Hardware

**Figure 5:**
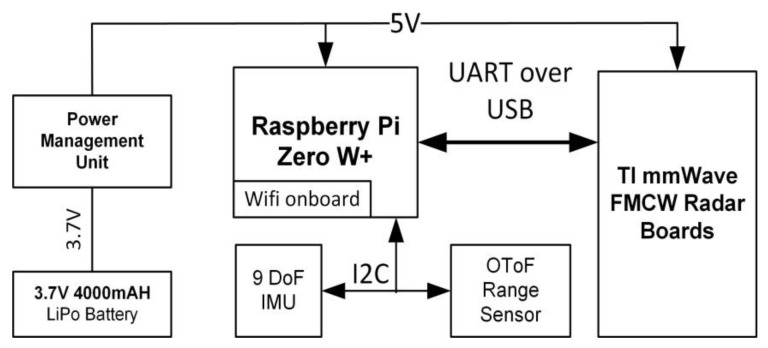
Hardware block diagram of smart-shoe prototype. The Raspberry Pi acts as the main command and control device, as well as a datalogger. The main sensor is the TI mm-wave radar, and the secondary sensors are a nine degree of freedom IMU and Optical Time-of-Flight range finder.

A prototype was built based on evaluation boards from Texas Instruments, the IWR6843ISK and the MWAVEICBOOST (block diagram in Figure 5); this radar system was selected due to its operating frequency in 60 GHz, which falls into the unlicensed part of the spectrum, making it ideal for new applications. Moreover, the 60 GHz band has high gaseous absorption in oxygen [112], which attenuates interference from other distant sources, making the system more robust. A final advantage of this band is that the bandwidth can be as high as 7 GHz. The IWR6843 has a bandwidth of 4 GHz, achieving a range resolution of 3.4 cm, and has three transmitters (Tx) and four receivers (Rx), enabling MIMO through multiplexing transmitters in time. The Tx antenna array is a sparse array, having one odd antenna in the line to enable elevation angle estimation, and the Rx array is a full array in line, enabling azimuth angle estimation. This MIMO configuration enables not only obstacle detection but 3D location with respect to the shoe. 3D obstacle detection would not be possible in this application with other methods such as conventional analog phased array, since they require more channels to achieve the same result, translating into more power consumption.

**Figure 6:**
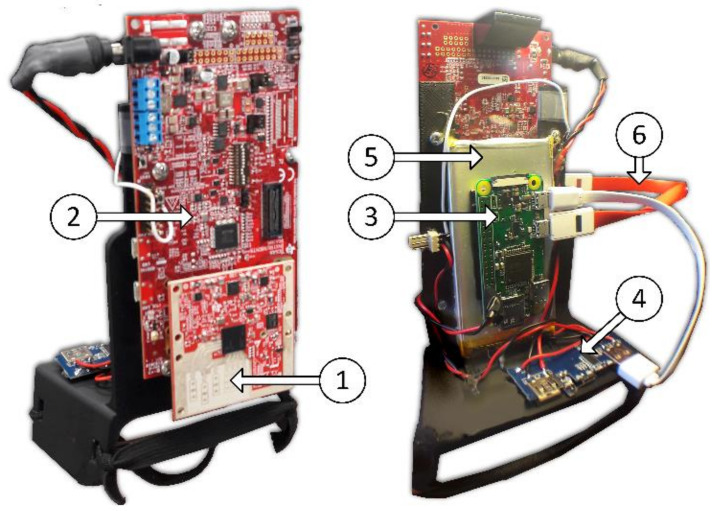
Smart-shoe instrumented foot-mount assembly. (1) IWR6843ASK Antennas and board, (2) MMWAVEICBOOST board, (3) Raspberry Pi Zero W+, (4) Power management board, (5) LiPo Battery and (6) USB Data cable. The mount was 3D printed and all the PCB boards and battery were fixed to it. The setup was mounted to the shoe using elastic bands.

A Raspberry Pi Zero W+ was selected as the control and data logging unit due to its reduced size and its Wi-Fi capability. Two additional sensors were included, an Inertia Measurement Unit with nine degrees of freedom (three accelerometers, three gyroscopes and three magnetometers) and an Optical Time-of-flight distance measurement sensor. The power supply unit consisted of a 4000 mA, 3.7 V Li-Po battery and a commercial USB battery managing circuit. All electronics were attached to a 3D-printed foot-mount shown in Figure 6.

### 5.2. Software

The IWR6843 runs a custom firmware [113] that performs time domain multiplexed multiple-input multiple-output FMCW radar signal processing. The three transmitters are cycled to obtain three radar cubes with four receiving channels, allowing the signal processing chain to first perform a range FFT and a Doppler FFT. Later, angle estimation is calculated via elevation and azimuth FFTs [114] and targets are detected using Cell Averaging Constant-False-Alarm-Rate (CFAR-CA) processing. This CFAR-CA produces a/one(?) point cloud per frame, and it is delivered through a serial output to the Raspberry PI.

**Figure 7:**
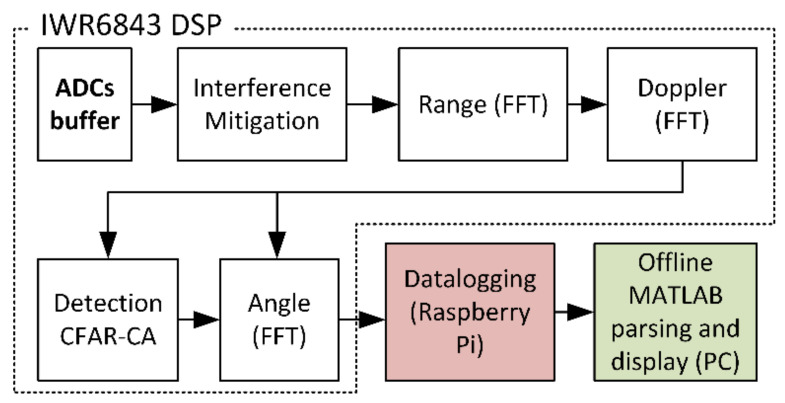
Software and digital signal processing chain block diagram. The four receivers’ intermediate frequency (IF) output is digitized by four analog to digital converters (ADCs) and then filtered to reduce interference. The signals are then processed by a chain of FFTs to find range, elevation and azimuth angles and, finally, they are converted to a point cloud by a CFAR-CA block. These data are then serially passed on to the datalogger and then parsed, post-processed and displayed in MATLAB.

The Raspberry Pi runs a suite of software developed in Python and PHP specifically for this application. The main python program is run upon power-up and is responsible for configuring and controlling the radar and receiving the data for storage in the file system within the SD card. This python program can spawn a series of independent python scripts that interact with the secondary sensors and store their data in a separate file in the same file system (Figure 7).

The Raspberry Pi also runs an Apache Server with PHP for command and data control through HTTP. The smart-shoe can be controlled by accessing a simple PHP-developed interface on a web browser by typing the IP address of the system. The PHP interface interacts with the main Python program allowing the user to start and stop the sensors and initiate data logging by the click of a button. The data can then be extracted from the data directory via the web browser onto a PC that runs an offline MATLAB parsing script for data visualization.

### 5.3. Single Stationary Obstacle Distance Measurement Experiment

An experiment was conducted to explore the distance measurement capability of the radar with a single, stationary, real-life obstacle: a piece of wood.

**Figure 8:**
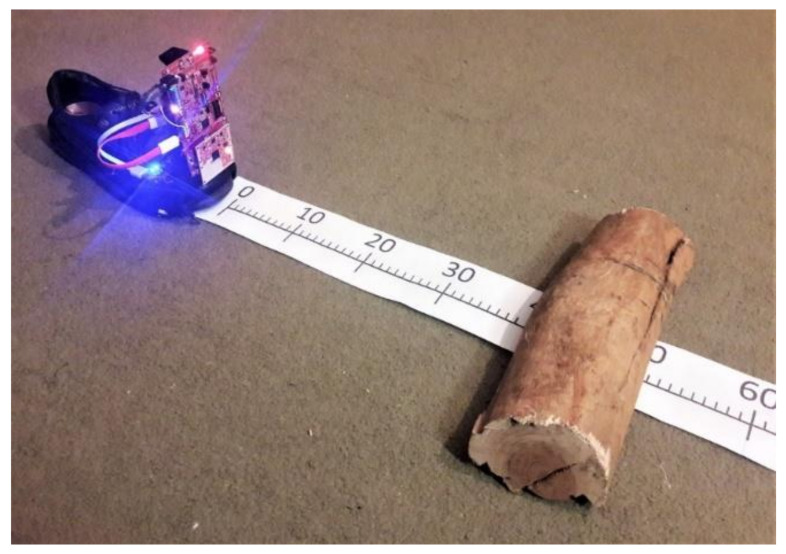
Experimental setup. A wooden obstacle was placed at different distances from the smart shoe prototype to characterize the radar sensor accuracy and detection capacity.

The smart shoe prototype was placed on a carpeted floor with a graded scale (cm; Figure 8). The target was a semi-cylindrical log, approximately 10 cm in diameter and 30 cm in length.

The log was placed on the 70 cm mark and then the smart shoe data-logger was started. After the initialization was finished, the log was moved towards the shoe from 70 cm to 0 cm in 10 cm steps, modifying its position every 10 s approximately.

Results were downloaded from the datalogger GUI to a PC and then parsed into MATLAB. A range limiting filter was applied to remove unwanted clutter, all detected objects beyond 1 m were discarded.

**Figure 9:**
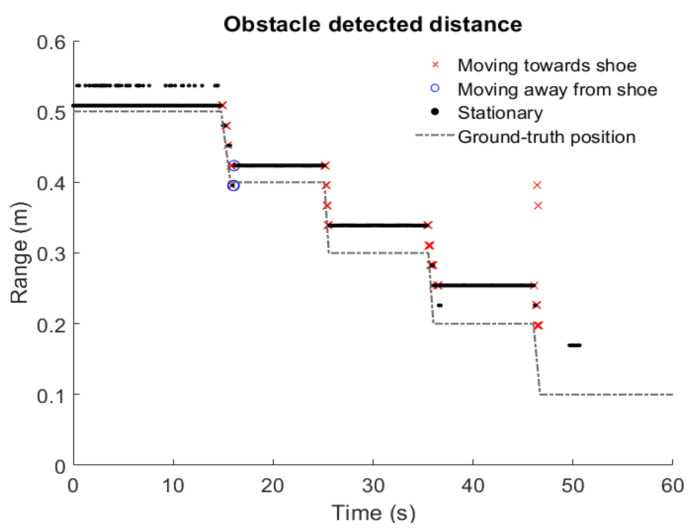
Experimental results. The radar was able to detect the obstacle at a distance between 10 cm and 70 cm. The accuracy improves as the obstacle is moved away from the radar. This effect could be caused by the near-field effects, where the incident reflections can no longer be considered parallel. A possible solution is to repeat this experiment several times to obtain a correction factor to affect the measurements, since the error seems to follow a linear trend.

The range of the detected object within the interest area was plotted in comparison to the ground truth position (Figure 9). Results indicate that the object was only detected within a range of 10 to 50 cm and that the error increases when the distance to the radar decreases. Causes of this error need to be investigated further, however, some hypotheses are that: (1) the target is significantly bigger than the wavelength (10 cm >> 5 mm) and bigger than the range resolution, therefore the grouping of the detected blips can introduce error into the distance measurement; (2) measurements are affected by near-field effects.

### 5.4. Detection of Staircase Steps Experiment

In order to investigate the possibility of detecting steps on a staircase and then determining if the person is successfully clearing them, an experiment was designed that involved wearing the smart shoe and going up and down the stairs several times.

Five trials involving going up and down one step of the staircase at different paces were recorded. In addition, two trials involving repeatedly colliding with the step were recorded. Results were filtered by range to eliminate unwanted returns from objects further ahead of the area of interest. The range of the object was calculated from coordinates with the following equation:

(1) $$r=\sqrt{x^{2}+y^{2}+z^{2}}$$

**Figure 10:**
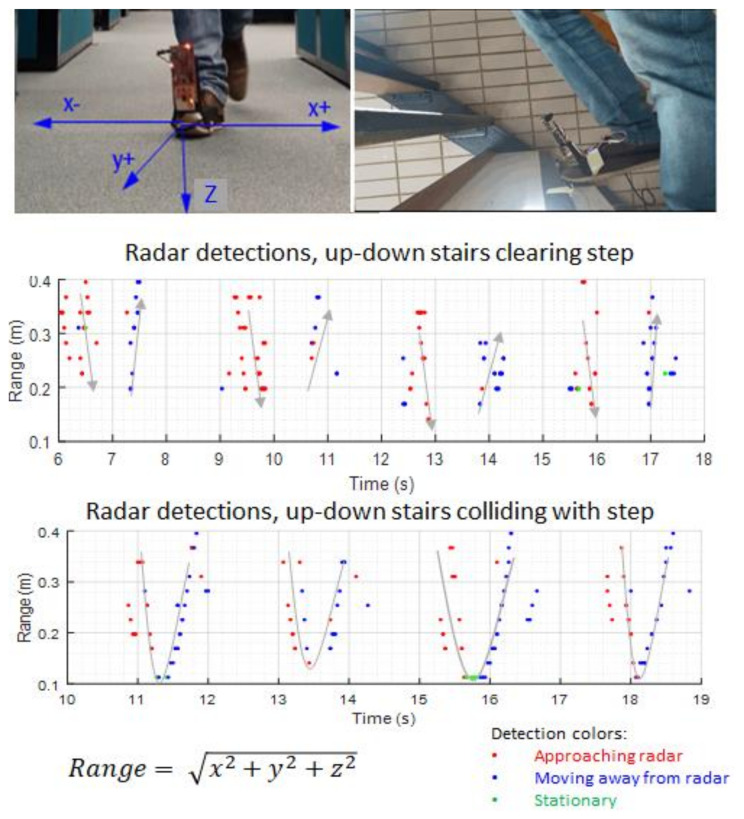
Experimental results. The radar was able to detect the staircase steps at a distance between 10 cm and 40 cm with the foot in movement towards and away from it. The points represent radar detections, and the colours represent direction of the Doppler speed estimation. Gray curves have been superimposed on the detection to highlight the difference in trajectories when the foot clears or collides with the step.

In Figure 10, each dot represents a detected object with range in the *y*-axis and Doppler speed direction displayed as colors and time on the horizontal axis. The reference system of coordinates can be seen as shown in the photo in the top left corner of the figure. Analyzing the figure, we can observe that the detected obstacles distance seems to match the trajectory of the foot going up and down the step, and the difference between the non-colliding and colliding trajectories is noticeable as shown by the gray curves adjusted to the point clouds.

Subsequently, a preliminary training of machine learning algorithms was conducted in MATLAB classification learner to preview the possibility of identifying different foot–obstacle trajectories. The data collected from the each of the experiments consisted of 20 steps up and down; that is to say, a total of 100 steps clearing and 40 steps colliding. The data were then sliced manually into single steps and classified in “clearing” and “colliding” arrays. Later, 80% of the organized data were fed to the classification learner as training. The remaining 20% were used as testing data. The MATLAB toolbox trained and tested 10 different classification models, including linear and quadratic support vector machines, discriminant analysis and nearest neighbour. The best results were obtained with a KNN Combination algorithm, reaching 97% classification accuracy.

### 5.5. Comparison of Wearable Radar in This Work with Previous Related Research

The radar system presented in this work explores the usage of a frequency band that had not been used for fall prevention before either in wearable or non-wearable form. A comparison of the specifications and capabilities of our sensor versus previous work is presented in Table 3.

## 6. Future Research and Emerging Applications

The successful detection and location of obstacles could allow for fall prevention to be carried out by feeding predicted foot trajectories calculated from the secondary sensors, such as the IMUs, and the location of objects detected by the radar to a machine learning algorithm that can classify the high-risk obstacles, then this output can be used to give timely feedback to the user to avoid tripping, for example by means of haptic feedback (vibrating device in the shoe) or a beeping sound. In order to achieve good prediction, the range and cross-range resolution of the radar system needs to be improved. This can be achieved by increasing the bandwidth of the signal and the number of transmit/receive elements. Also, the improvement of the gain and radiation pattern of the antenna system will allow for better obstacle detection, increasing the RCS of the obstacles to detect.

Moreover, processing speed and data delivery speed are key to inform the user in a timely manner. Swing time in older adults is under 0.5 s [115] and feedback would need to be provided during or before this phase to account for reaction time. Current technology, such as the one used in our proof-of-concept, can output an array of detected obstacles at a rate of 30 Hz maximum, which is fast enough, however, the signal must still be captured and post processed by the MCU to predict the tripping risk. Significant research is needed in these post processing algorithms, feedback mechanisms and user reaction times to achieve this goal.

**Figure 11:**
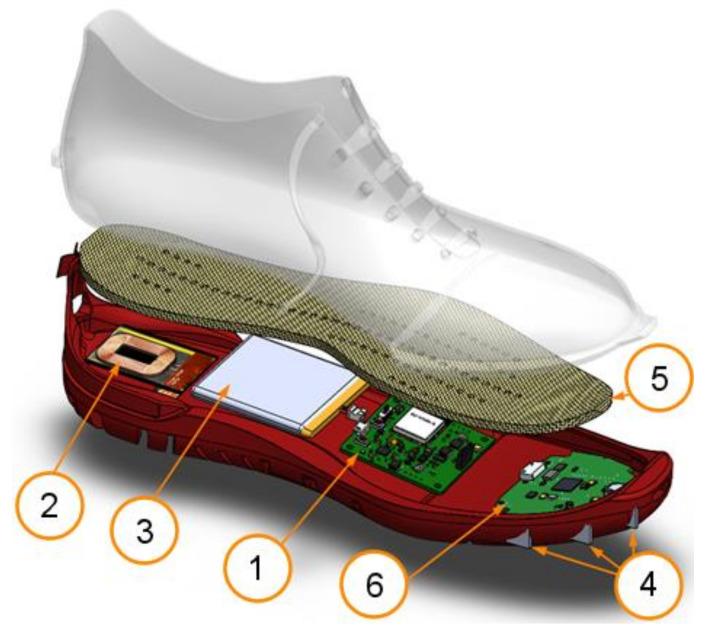
Proposed Smart-Shoe Implementation Concept. (1) MCU + IMU + wireless communications; (2) inductive charging; (3) LiPo battery; (4) antenna array; (5) pressure sensing insole; (6) radar electronics. Wiring is not shown in the schematic for the sake of clarity.

Figure 11 shows a concept design of the full system of a smart shoe, including the radar electronics and conformal antenna array, a processing unit, a power supply and secondary sensors. Further miniaturization and optimization of the electronics and antennas need to be achieved in order to embed all of the parts in the shoe. Another challenge is to develop the machine learning algorithms and software that can be fully run onboard.

Even though the most common applications of gait analysis are clinical, such as fall prevention or treatment evolution tracking, several other markets have emerged in recent years. Sports and defence related applications, for example, present research and development opportunities, and a radar-based smart-shoe system for gait analysis could be easily adapted to these requirements. In defence applications, they could provide a useful insight into a front-line combatant’s health and assist in the evolution of skills in training and combat situations, providing extra data about the environment that would not be possible to obtain with inertia measurement units or pressure sensors. In the field of sports, radar-enabled smart shoes could provide an affordable cutting-edge tool for assessing performance and analyzing skills, for example tracking the interaction between a football and the sportsman’s foot.

## 7. Conclusions

A review of wearable and radar sensors for gait analysis and fall prevention reveals that, even though the utility of wearable sensor-based gait analysis is becoming widely recognized, we are only beginning to realize its potential. Fall prevention in everyday living conditions is still a largely unexplored area, and the development of new tools would be highly beneficial for researchers, clinicians and patients.

Radar could be useful for measuring gait parameters and performing fall risk-assessment using statistical methods and could also be used to monitor obstacles in real time.

A preliminary prototype for a smart shoe for trip hazard detection based on commercial components was designed, built and experimentally tested. Experimental results suggest that fall prevention and gait analysis can be achieved; nevertheless, further research is necessary to advance the technology for commercial application. Future work should focus on improving the range and angle resolutions by adding more channels and increasing the bandwidth and designing and assembling a more integrated and outdoor-proof prototype. This means embedding all the hardware in the sole and sides of the shoe and upgrading the software for standalone operation. Tailored antenna elements and array shapes should be designed to be conformal to the shape of the shoe and to maximize the detection capabilities of the radar. This will enable easier and more comfortable use by the wearer, ensuring that it will not force the subject to modify his/her gait while wearing it.

## Figures and Tables

**Table 1:** Feet Wearable Gait Analysis Sensors.

Sensors	Details	Strengths	Weaknesses	References
Inertia measurement unit (IMU)	3-axis accelerometry and gyroscopic data can be obtained and processed to estimate spatiotemporal parameters. Foot clearance can be estimated with integrational methods although is very inaccurate. They are used on 18 of 35 of the studies surveyed	Inexpensive Signals are easy to measure	Drift Non-direct parameter estimation Placement dependent	[8,20,39,48,49,50,51,52,53,54,55,56,57,58,59,60,61,62,63]
Force sensitive resistors (FSR)	Vertical ground reaction forces can be measured and used to estimate spatiotemporal parameters. They are used on 6 of 35 of the studies surveyed	Inexpensive Signals are easy to measure	Sometimes temperature dependent Vertical force sensing only	[8,9,48,63,64,65,66,67]
Pressure sensors	They measure vertical ground reaction forces and plantar pressure distribution. Can be used to perform gait classification depending on the distribution and to estimate temporal parameters. They are used on 5 of 35 of the studies surveyed	Overcome temperature drift problems of FSRs	Normally several sensors are needed. Vertical force sensing only Multiple channel processing is needed Bulkier than FSRs	[39,51,56,59,68,69,70]
Optical Time of Flight (OToF)	Foot clearance measurement They are used on 2 of 35 of the studies surveyed	Good accuracy	Fails to measure when the walking surface is black or transparent	[61,62]
Ultra-sonic	Foot clearance measurement They are used on 2 of 35 of the studies surveyed	Inexpensive Easy data extraction	Bulky High energy consumption	[49,50]
Radar and RF	Foot clearance measurement and obstacle detection They are used on 2 of 35 of the studies surveyed	Good position change measurement accuracy. Obstacle detection capability	Heavy signal processing High energy consumption	[71,72,73]
Piezo-electric Bend	Temporal event detection (Toe-off/Heel strike) They are used on 1 of 35 of the studies surveyed	Inexpensive	Not accurate Limited mechanical lifespan	[74]

**Table 2:** Radars in Gait Analysis and Fall Detection/Prevention.

Radar Type	Freq.	Functionality/ Parameters Measured	Signal Processing Type	Wearable	References
Continuous wave	10.5 GHz	Identified features in the doppler radar signature from foot, leg, and thorax using SFTF and model extraction. Parameter extraction not reported.	Short-time Fourier transform, chirplet transform	No, Tripod-mounted	[79,80]
Pulse-Doppler range control radars	Not reported	Fall detection. Reported classification rate between 0.91 and 0.97.	SVM and kNN to detect falls based on the extracted Mel frequency cepstral coefficients (MFCC) features	No, 2x Placed on floor	[81]
Pulse-Doppler range control radar	5.8 GHz	Fall detection. Reported accuracies vary between 77.0% to 93.0% depending on wavelet type and classifier type.	Wavelet transform. Two-stage processing: prescreening and classifying (MFCC and TS)	No, Ceiling-mounted	[82]
Pulse-Doppler	5.8 GHz	Gait parameters extraction from doppler signature: Gait velocity and Stride rate. No numerical results reported. Figures indicate good matching between motion capture system (gold standard) and radar, when subject has normal gait, and regular performance when the subject has a condition such as a stroke or Parkinson’s.	Short-time Fourier transform, spectrogram filtering and peak detection	No, 2x Placed on floor	[83]
Wideband FMCW	3–10 GHz	Gait speed. Average error reported to be 7.3% for slow speed and 12% for normal speed.	Short-time Fourier transform	No, Tripod-mounted	[84]
Narrowband FMCW	24 GHz	Gait speed. Average error reported to be 10.33% for slow speed and 5.80% for normal speed. Very short detection range reported.	Short-time Fourier transform		
FMCW	24 GHz	Gait speed. Maximum accuracies reported are 86% for high speed, 81% for normal speed and 74% for low speed.	Not reported. Commercial radar solution with human tracking software used.	No, Tripod-mounted	[85]
Stepped frequency continuous wave (SFCW)	5.8–7.0 GHz	Fall detection. Reported accuracy of 94.3% for fall and fall-like events.	Inverse Fast Fourier Transformer (IFFT) for range profiling. Movement classification using machine learning Least Squares Support Vector Machines (LS-SVM)	No, Tripod-mounted	[86,87]
Micro-Doppler	Ka-band	Long Range Front-View Gait Recognition of People. Classification accuracy 40 to 80%, angle-of-arrival dependent.	Short-time Fourier transform, Stride rate is extracted and used through a proprietary algorithm to classify between different individuals.	No	[88]
Pulse-Doppler + Kinect Cameras	Not reported	Real-time monitoring for fall detection with sensor fusion. 98% detection rate. Most of the success reported is due to the Kinect cameras, radar correlations with gold standard reported to be poor.	Not reported.	No Ceiling mounted	[89]
Continuous wave (NI-USRP 2922)	4 GHz	Gait classification, fall detection. Reported success rate 83.35%	Linear predictive coding coefficients (LPC), discrete-cosine coefficients (DCT) and support vector machines for classification.	No	[90]
Dual pulse-Doppler range control radar (commercial RCR-50)	5.8 GHz	Quantitative gait measurement, Reported step time ICC of 0.97. Gait speed ICC of 0.99	Short-time Fourier transform plus tailored algorithms	No, Mounted a torso level and at foot level	[91]
Continuous wave	10.525 GHz	Human gait recognition (classification) vs. animals. Reported successful classification rate of 88%	Short-time Fourier transform plus tailored algorithms	No, Tripod mounted	[92]
2 x FMCW Doppler (IVS-162 DRS)	24.25 GHz	Gait velocity measurement during rehabilitation of patients. Numeric results were presented but not compared to any gold standards.	Short-time Fourier transform	No, Mounted on walker	[93]
Dual-frequency continuous wave	7.9 GHz	Terrain relative velocity measurement. Maximum distance error in lab (not foot mounted) was 1.3 mm and minimum detectable velocity 0.19 mm/s.	I-Q demodulation and phase changes for distance calculation.	Yes, under shoe heel	[71,72]
FMCW Commercial InnoSenT IPS-154	24.125 GHz	Qualitative analysis of Doppler spectrum of different gait phases including walking and going upstairs. No quantitative measurements were reported.	Short-time Fourier transform	Yes, Foot and/or ankle mounted	[94]
1 × FMCW radar + 16 Ultrasonic sensors	Not reported	Visually impaired aid for navigation. No information about performance was disclosed.	Not reported	Yes, Chest band	[95]
FMCW	23.79–24.35 GHz	Fall prevention through obstacle detection. Feasibility of obstacle detection demonstrated with tin can. Numerical results for distance measurement report 1.76 cm average error and 4.5 cm worst case scenario between 40 and 240 cm.	FFT and normalization	Yes, Shoe mounted	[73,96]

**Table 3:** Comparison of wearable radar in this work with previous related research.

References	Radar Type	Freq. and BW	# TxRx	Standalone System	Batt. Life	Obstacle Detection	Signal Processing Type
[71,72]	DFCW under shoe heel	7.9 GHz N/A	1 1	No Signal acquisition and processing are external	N/A Wired	No, terrain velocity was measured.	Phase calculation. Performed on external computer
[94]	FMCW InnoSenT IPS-154 foot and/or ankle mounted	24.13 GHz Not rep.	1 1	No Signal acquisition and processing are external	N/A Wired	No, Qualitative analysis of Doppler spectrum of different gait phases.	Short-time Fourier transform. Performed on external computer
[95]	1 x FMCW radar on chest band	Not rep. Not rep.	1 1	No Signal acquisition and processing are external	N/A Wired	Yes, Visually impaired aid for navigation. No information about performance was disclosed.	Not reported.
[73,96]	FMCW shoe mounted	24 GHz 560 MHz	1 1	No Signal acquisition and processing are external	N/A Wired	Yes, metallic (conductive) only. Numerical results for distance measurement report 1.76 cm average error and 4.5 cm worst case scenario between 40 cm and 240 cm.	FFT and normalization. Performed on external computer
This work + previous [108,109]	MIMO FMCW TI. IWR6843 shoe mounted	62 GHz 4 GHz	3 4	Yes, for data collection including obstacle detection. Classification using MATLAB postprocessing requires external computer	4 h	Yes, conductive, and non-conductive. Numerical results for conductive were not analyzed. Results for non-conductive show detectability between 5 cm and 60 cm away from target. Data without correction show worst case scenario error to be 8 cm, but this could be reduced with linear regression.	Range-FFT, Doppler-FFT, CFAR-CA and Angle-FFT. All performed onboard. Classification requires external computer.

## Data Availability

Not applicable.
